# Prevalence of small testicular hyperechogenic foci in subgroups of 382 non‐vasectomized, azoospermic men: a retrospective cohort study

**DOI:** 10.1111/andr.12291

**Published:** 2017-01-06

**Authors:** J. Fedder

**Affiliations:** ^1^ Centre of Andrology and Fertility ClinicOdense University HospitalOdenseDenmark

**Keywords:** azoospermia, cryptorchidism, cytokeratin‐18, Klinefelter's syndrome, testicular hyperechogenic foci, testicular microlithiasis

## Abstract

Testicular hyperechogenic foci (THF) are associated with Klinefelter's syndrome, cryptorchidism, infertility, and testicular germ cell neoplasia. The aims of the study were to evaluate THF in relation to etiology of azoospermia and to Sertoli cell dysfunction. The structures inside the scrotum of consecutive non‐vasectomized, azoospermic were examined by ultrasonography, and hormone (FSH, LH, testosterone, and prolactin), and genetic analyses (karyotype, Y microdeletions, and CFTR mutations) were performed. At testicular ultrasonography, patients were graduated into: pronounced THF (>7 THF per transducer field), distributed universally (uTHF) or collected in plaques (pTHF), borderline THF (bTHF; 3–7 THF per transducer field), or no THF (<3 THF per transducer field). Diagnostic testicular biopsy was taken open or with TruCut needle (14G). THF status was sufficiently described in 382 of 449 potential participants, and testicular histology was available in 300 cases. Presence of ultrasonographically detectable THF was compared to presence of testicular microlithiasis (TM) detected histologically. Sertoli cell dysfunction was investigated in a subgroup using a three‐stage immunoperoxidase technique for detection of cytokeratin‐18 (CK‐18). The prevalence of THF was 13.4%. uTHF was found in 11 men (2.9%), the pattern was bilateral in four while other four had bTHF in the other testis. pTHF was detected in eight cases (2.1%), and except for one case with Klinefelter's syndrome, pTHF was in all cases occurring unilaterally. bTHF was detected in 32 cases (8.4%), bilaterally in 17 (53%). Pronounced THF was significantly associated with testicular malignancy. CK‐18 was detected in more azoospermic men with sperm production in ≤50% seminiferous tubules than in azoospermic men with spermatogenesis in ≥90% of seminiferous tubules and normal controls (*p* < 0.05). Unfortunately, TM detected histologically was not detected in any patient expressing THF, and neither THF nor TM was detected in any of the patients examined for CK‐18. Sertoli cell dysfunction was not associated with testicular microlithiasis or hyperechogenic foci.

## Introduction

In 1987, Doherty *et al*. (1987) described a 23‐year‐old man, who at age 10 had an orchiopexy for cryptorchidism. By ultrasonography, a pattern of innumerable tiny bright echoes (‘white spots’) diffusely and uniformly scattered throughout the testis tissue was found. The atrophic and previously cryptorchid testis was removed by an inguinal orchiectomy. By histological evaluation, no tumor was found, but calcific concretions were present in 30–40% of the seminiferous tubules, and the histologic diagnosis was testicular microlithiasis (TM).

As this report was published, several studies evaluating the prevalence of men with such white spots (testicular hyperechogenic foci = THF) in the testis tissue and its association with pathological, andrological conditions, have been performed. The evidence documenting that THF in all cases represents TM seems insufficient, and presence of such white spots in the testis in this study is termed THF, although usually termed TM in the literature. Usually >5 or ≥5 THF 1–3 mm of size in one transducer field (Backus *et al*., 1994 & Peterson *et al*., 2001) or in one testis (Goede *et al*., 2009, 2010) is considered pathological. The limit of five THF per transducer field has been used uncritically as the limit for abnormally high numbers.

Histologically detected microliths were initially described by Nistal *et al*. (1979). They are located within the seminiferous tubules and consist of a core of hydroxyapatite (Smith *et al*., 1999). This core is surrounded by concentric layers of collagen fibers surrounded by material rich in glycogen (DeJong *et al*., 2004). Vegni‐Talluri *et al*. (1980) has suggested that TM originates from degenerated intratubular cells not phagocyted by the Sertoli cells. Thus, it may be hypothesized that the background of TM could be defective spermatogenesis or dysfunction of the Sertoli cells (Vegni‐Talluri *et al*., 1980).

Using the definition of >5 THF per transducer field, prevalences of many abnormal THF in healthy young men have been found to be 2.4–5.6% (Peterson *et al*., 2001 & Serter *et al*., 2006) depending on ethnicity and geographical origin of USA, showing the highest prevalence in black men and men from southeastern states. In the majority of cases, THF occurs bilaterally (Peterson *et al*., 2001; DeGouveia Brazao *et al*., 2004; Serter *et al*., 2006 & Cooper *et al*., 2014).

Several studies have demonstrated or suggested an association between THF and pathological andrological conditions such as Klinefelter's syndrome (KS) (Martinez‐Valls *et al*., 2003 & Fedder *et al*., 2015), cryptorchidism (Nistal *et al*., 1979; Riebel *et al*., 2000 & Goede *et al*., 2010), and infertility (Aizenstein *et al*., 1998 & von Eckardstein *et al*., 2001). Furthermore, presence of THF has been associated with (increased risk of) intratubular germ cell neoplasia (IGCN) (van Casteren *et al*., 2009).

However, in almost all studies on THF (usually termed TM), the diagnosis is based solely on ultrasonographic detection of white spots in the testicles (van Casteren *et al*., 2009 & Goede & Hack, 2012). Histological examination is with few exceptions limited to evaluation of spermatogenesis and presence of testicular germ cell neoplasia (TGCN) without description of histological detectable microliths in the testis tissue. In addition to a few case studies, only Holm *et al*. (2003) evaluated ultrasonographically detectable THF and histologically detectable TM on the same material. However, it is not evident how often TM was found in patients with THF (Holm *et al*., 2003). As THF might represent other conditions than TM as seen after, for example, operations on the testicles (Fedder *et al*., 2016), examination of the associations between THF and TM in larger studies is greatly required.

Published data represent a huge variation in numbers and distributions of THF, and the significance of a low number of THF (borderline cases) has yet to be discovered. It seems highly relevant to evaluate the prevalence of THF in a cohort of men with azoospermia, the most manifested condition of male infertility. Azoospermia has an extensive variety of etiologies (Fedder *et al*., 2004). In a previous study, a probable cause of azoospermia could be identified in 78% of 100 azoospermic, consecutively included men (Fedder *et al*., 2004). Therefore, it is relevant to look for potential associations of THF with specific subgroups of azoospermia.

To answer the hypothesis that immature or dedifferentiated Sertoli cells might cause TM, we included results from a minor series of consecutive participants among these azoospermic men examined for the presence of the Sertoli cell differentiation marker cytokeratin‐18 (CK‐18) by immunohistochemistry (Franke *et al*., 2004). An association between CK‐18 and THF/TM might contribute to explain the mechanisms in THF/TM formation. Expression of CK‐18, which in normal men is found only in fetal Sertoli cells, is frequently found in Sertoli cells from adult men with cryptorchidism or TGCN (Rogatsch *et al*., 1996). Such men also have an increased frequency of THF.

The aims of the study were to evaluate:

- Whether presence of THF correlated with histological detection of TM,
- The possible associations of THF patterns and intensities with different categories of azoospermic men, and
- The possible associations between presence of cytokeratin‐18 (CK‐18) and ultrasonographically detected THF and histologically detected TM.

## Materials and Methods

The diagnosis azoospermia was based on examination of at least two ejaculates including centrifugation and microscopic evaluation of the pellet. Since 1997, we have performed an examination program as described by Fedder *et al*. (2004) for all men referred with azoospermia on a non‐vasectomy basis. The program was initiated in the Fertility Clinic, Horsens‐Braedstrup Hospital, and from May 2011 in an extended version carried on in the Centre of Andrology / Fertility Clinic, Odense University Hospital. All clinical examinations, including ultrasonography and testicular biopsy, were performed by the same specialist.

All included men were examined clinically, including body composition and body proportions, hair distribution, and scrotal examination. Basis hormone analyses included: FSH, LH, testosterone, TSH, and prolactin, and genetic analyses included karyotype, Y microdeletions, and CFTR mutations (Fedder *et al*., 2004). Two specific sequence‐tagged sites (STSs) were used for detection of deletions in each of the AZFa, AZFb, and AZFc regions. Furthermore, the patients were examined for deletion in the SRY region. Routinely, the patients were examined for 32 CFTR mutations in the exons and for mutations in IVS8 5/7/9T (intron 8).

The program was carefully performed with the purpose to subclassify the azoospermic men into different etiological categories. Although this program does not represent a prospective study with testicular ultrasonography as primary outcome, testicular ultrasonography was systematically performed as part of the program.

### Ultrasonography of the scrotum

Ultrasonographic investigation of the scrotal contents was performed using Siemens equipment, including 7.5 MHz linear transducers. Testicular volumes were calculated as: π/6 × testis length × (testis diameter)^2^ (Behre *et al*., 1989), and the echogenicity of the testicular tissue was evaluated.

More than seven THF in one transducer field is in this study considered as clearly pathological. However, THF patterns show a considerable variation according to the amount as well as the distribution of THF. Testicles with 3–7 THF were considered as belonging to a ‘borderline’ subgroup (bTHF). Men with pronounced THF were subdivided into two subgroups, considering men with universally distributed THF (uTHF) as one subgroup and men with THF located in ‘plaques’ (pTHF) as another subgroup.

### Biopsy procedure

All azoospermic men without KS and hypogonadotropic hypogonadism were offered testicular biopsy, unless the couples had in advance decided to use donor semen (and the ultrasonography showed no THF).

Following application of lidocaine, 200–400 mg, into the upper scrotum on each side of the spermatic cord, the testicular biopsy was taken either as a traditional open biopsy detaching a specimen of testicular tissue bulging out through an incision on the *Tunica albuginea* (Fedder *et al*., 2004) or more often as one to three TruCut biopsies (Ch 14) (Fedder, 2011). The first 120 men had open biopsies, while the remaining men had TruCut biopsies. If living spermatozoa were found in the first testicle, and the other testicle appeared normal without THF, testicular biopsy was only performed unilaterally. In the remaining cases, testicular biopsy was performed bilaterally provided the man had two testicles. Each TruCut biopsy has a volume of 24 mm^3^ (Fedder, 2011). With each technique, areas with at least 100 seminiferous tubule sections were usually available, and therefore, the histological diagnosis was representative and independent of the biopsy technique chosen.

### Immunohistochemistry

In a prospective substudy including a consecutive series of 33 azoospermic men also included in the main study, and 7 normal controls undergoing vasectomy, the maturity of the Sertoli cells was determined based on detection of CK‐18 by immunohistochemistry. A piece of the diagnostic testicular testis biopsy was snap‐frozen in liquid nitrogen.

Cryostat microtome sections, 8‐μm thick, were cut and stored at −70 °C until fixed in paraformaldehyde, 2% (5 min) and stained using a three‐stage immunoperoxidase technique. The sections were preincubated with 1% BSA in COON's for 1 h, and endogenous biotin was blocked using Vector SP2001. CK‐18 was detected by a mouse monoclonal antibody (Santa Cruz Biotechnology, cat#sc‐6259) (1 h, room temp). A mouse immunoglobulin G_1_ (Pharmingen 33811A) was used as control. Thereafter, the sections were incubated with biotinylated donkey anti‐mouse antibody (Jackson, 715‐066‐150, 1 : 400, 45 min). Horseradish peroxidise–avidin biotin complex (HRP‐sABC, DAKO, K 0377) was used for identification of the antigen–antibody binding sites, and the peroxidase activity was visualized using a 0.05% solution of diaminobenzidine tetrahydrochloride (DAB, Sigma D‐5637, 15 min). Counterstaining was performed with Mayer's hematoxylin (5 min). The stained sections were mounted with Aquamount BDH 362262H and read blindly.

COON's buffer with 1% BSA was used as blocking and washing buffer up to and including the secondary antibody layer. TRIS 0.005 m, pH 7.6 was used for the third layer and the substrate including wash before application of these two layers. Following substrate and hematoxylin application, tap water was used for wash.

### Statistics

Associations between THF patterns and testicular neoplasia and between CK‐18 and spermatogenesis were evaluated using Fisher's exact test.

### Approvals

The study was approved by the Danish Data Protection Agency (15/17627) and the Danish Health and Medicine Authority (3‐3013‐1088/1). The prospective immunohistochemical substudy with CK‐18 was further approved by the Data Protection Agency and the local Scientific Ethics Committee (Vejle og Fyns Amter; J.nr. 97/258 PMC).

## Results

During the period November 1st, 1997–April 30th, 2015, 449 non‐vasectomized, azoospermic men were referred for examination. Several men referred with azoospermia were diagnosed as having cryptozoospermia (Ben‐Ami *et al*., 2013) and were not included. Although all azoospermic men went through the full evaluation program, the presence of small testicular hyperechogenic foci was only sufficiently described in 382 patients (33 ± 6 years of age; Table 1), and the remaining 67 therefore were excluded for further analysis. Only the 382 men with a known THF status were included in the further study.

**Table 1:** Levels of testicular hyperechogenic foci (THF) distributed according to etiological subgroups of azoospermia

Primary diagnosis	Secondary diagnosis	<3 THF	3–7 THF borderline	>7 THF, plaques	>7 THF, universal
47, XXY (Klinefelter)	None	19	10	2	1
	Crypt., persisting	1	–	–	1
	Crypt., requiring operation	1	2	–	1
	Crypt., requiring hormone treatment	1	1	–	–
	Crypt. with spontaneous testicular descent	1	1	–	–
	CFTR mutations	2	–	–	–
Y microdeletion	None	12	3	–	–
	Crypt. requiring operation	1	–	–	–
	Crypt. with spontaneous testicular descent	3	–	–	–
	CFTR mutations	1	–	–	–
Crypt., persisting	None	4	1	–	–
	CFTR mutations	1	–	–	–
	Ejaculatory disorder	1	–	–	–
Crypt. requiring operation	None	42	6	1	–
	CFTR mutations	5	–	1	–
	Hypogonadotropic hypogonadism	1	–	–	–
	History of orchitis or genital tract infection	1	–	–	–
Crypt. treated with hormones	None	1	1	–	–
	CFTR mutations	1	–	–	–
	Hypogonadotropic hypogonadism	1	–	–	–
Crypt. with spontaneous testicular descent	None	13	1	–	–
	CFTR mutations	1	1	–	–
CFTR mutations	None	23	–	1	–
	History of orchitis or genital tract infection	2	–	–	1
History of orchitis or genital tract infection	None	14	1	–	1
Hypogonadotropic hypogonadism	None	2	1	–	–
Ejaculatory disorders	None	11	–	1	–
Unknown etiology		165	3	2	6

**Figure 1:**
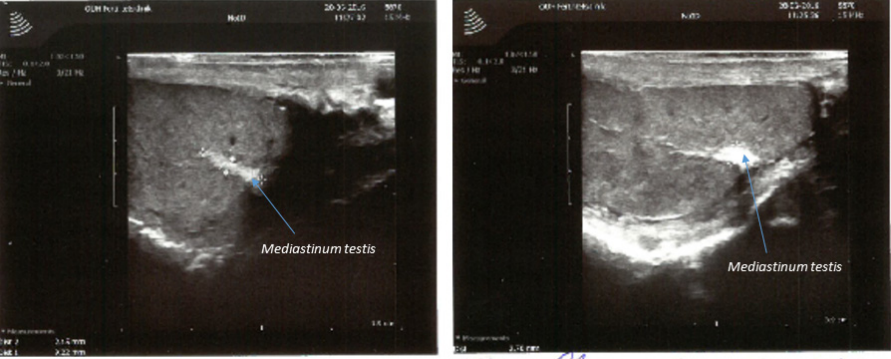
In normal testicles hyperechogenic, wedge‐shaped areas representing the *Mediastinum testis* are seen by ultrasonographic examination. [Colour figure can be viewed at wileyonlinelibrary.com].

**Figure 2:**
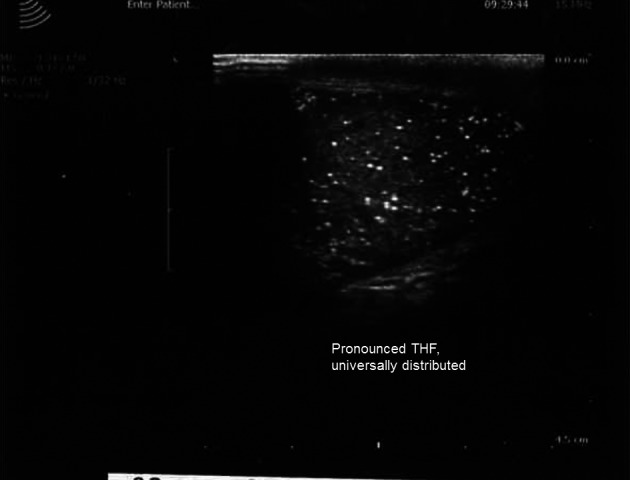
Universally distributed, pronounced THF in a 19‐year‐old man with azoospermia and only one detectable testis. Testicular spermatozoa from minor areas with apparently normal spermatogenesis were cryopreserved before the man was treated for intratubular germ cell neoplasia.

**Figure 3:**
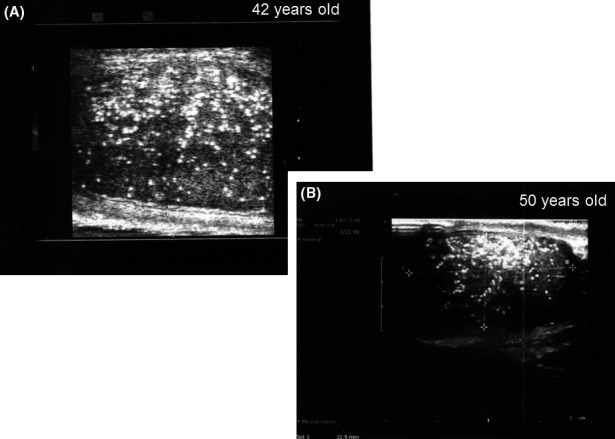
Patterns of universally distributed, pronounced THF in an azoospermic man, when he was 42 (A) and 50 years old (B). No malignancy was detected histologically.

**Figure 4:**
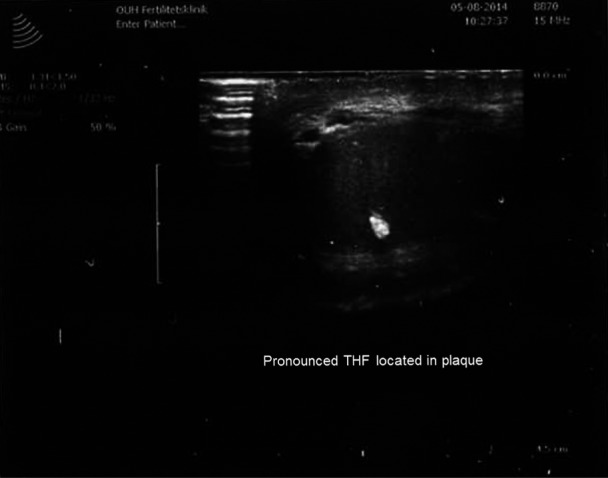
Pronounced THF located in five small plaques in a testis of a 22‐year‐old man with azoospermia. Histologically, he had intratubular germ cell neoplasia.

As shown in Fig. 1, normal testicles often show a wedge‐shaped hyperechogenic area representing the *Mediastinum testis*. uTHF (Fig. 2) was found in 11 men (2.9%). In four of these (36%), uTHF was found bilaterally. In four cases, including three with KS, bTHF was detected in the contralateral testis. The author had the opportunity to examine one of the men with uTHF when he was 42 years old and again when he was 50 years old. The THF patterns were very similar suggesting that the condition may remain unchanged over time (Fig. 3). A testicular TruCut biopsy taken after the last ultrasonography showed no signs of (pre‐)malignancy. pTHF (Fig. 4) was detected in eight cases (2.1%), and except for one KS man, the condition was in all cases unilaterally. bTHF was detected in 32 cases (8.4%), bilaterally in 17 (53%). Unilateral bTHF was found in a further four cases with uTHF in the other testis, and these cases were categorized as having uTHF. Microcalcifications were not detected histologically in testicular biopsies from any of the patients with ultrasonographically detected THF.

A frequent overlapping between the most relevant etiological groups was found, for example, 10 (23%) of the 44 men with KS and four (20%) of the men with Y microdeletions had a history of cryptorchidism. Of 40 men with CFTR mutations, four had THF. However, three of these four men had additional etiological factors, two had cryptorchidism and one had an ejaculatory disorder (Table 1).

Some diagnoses, for example, cryptorchidism, are categorized as secondary diagnosis in men with KS or Y microdeletions. When cryptorchidism was the only pathological andrological finding, this was categorized as primary diagnosis in Table 1. In azoospermic men with competing diagnoses, diagnoses causing primary testicular defects were ranked higher (more important) than diagnoses causing azoospermia on an obstructive basis.

Five of the KS men had pronounced THF, three (7%) had uTHF and two (5%) had pTHF, while 14 (32%) showed bTHF. Of 101 men with a history of cryptorchidism, two (2%) had uTHF, two (2%) pTHF, and 14 (14%) bTHF. The power of the study is not strong enough to meaningfully compare different subgroups of men with histories of cryptorchidism. All four men with pronounced THF were found among 73 (64 + 9) men operated on for or having persisting cryptorchidism.

bTHF occurred with a high prevalence in men with KS who all—as expected—had small testicular volumes. The prevalences of uTHF and pTHF were low, and as the conditions were distributed on different etiological categories, the power of this study cannot bear an evaluation of the association between THF and testicular volume.

Considering the association between THF (u+p+b) and impaired spermatogenesis of any kind (Sertoli cell only, maturation stop, or testicular atrophy), the sensitivity was 14.3% and the specificity 98.6%, showing that almost no men with normal testicular histology had THF. The positive predictive value of THF (u+p+b) to detect impaired spermatogenesis was 97.1% and the negative predictive value was 25.6%, showing that men with THF usually had impaired spermatogenesis in this study.

One of 266 men without THF had IGCN. Compared hereto the frequencies of TGCN and IGCN in men with uTHF was one of six (*p* = 0.04), in men with pTHF one of seven (*p* = 0.05), and in men with bTHF one of 21 (*p* = 0.14) (Table 2). In this study, positive and negative predictive values of THF to predict germ cell neoplasia (GCN) were 8.8% and 99.6%, respectively, suggesting that GCN seldom occurs in men without THF.

**Table 2:** The few azoospermic men with tumors and malignancy. Only the 300 patients having taken a testicular biopsy for histological examination are included in this table

THF pattern	History	Diagnosis	Treatment	Frequency in the subgroup	Significance level
uTHF (>7 THF elements, universally distributed)	Only one detectable testis	Intratubular germ cell neoplasia	Cryopreservation of testicular spermatozoa	1/6	*p* = 0.04
			Radiation, 16 Gy/8 fractions		
		Minor foci with sperm production			
pTHF (>7 THF elements, collected in plaques)	No specific andrological problems	Intratubular germ cell neoplasia	Orchiectomy. Ejac. spermatozoa one year after op.	1/7	*p* = 0.05
	Klinefelter's syndrome	Sertoli cell – Leydig cell tumor?	Unilat. Orchiectomy		
bTHF (3–7 THF elements)	Bilateral cryptorchidism in childhood (hCG)	Intratubular germ cell neoplasia	Orchiectomy	1/21	*p* = 0.14
No THF (<3 THF elements)	Anejaculation following myelomeningocele	Seminoma	Orchiectomy	1/266	

In one particular case, a 19‐year‐old man with uTHF and only one detectable testis, IGCN was found (Fig. 2). As a boy, he was extensively examined, including laparoscopy, but the testicle was not found. Because the young man had azoospermia, it was not possible to cryopreserve the ejaculated spermatozoa prior to operation. However, a large number of living and morphologically normal spermatozoa were isolated from foci with sperm production in the present testis. These spermatozoa were cryopreserved before radiation therapy.

**Figure 5:**
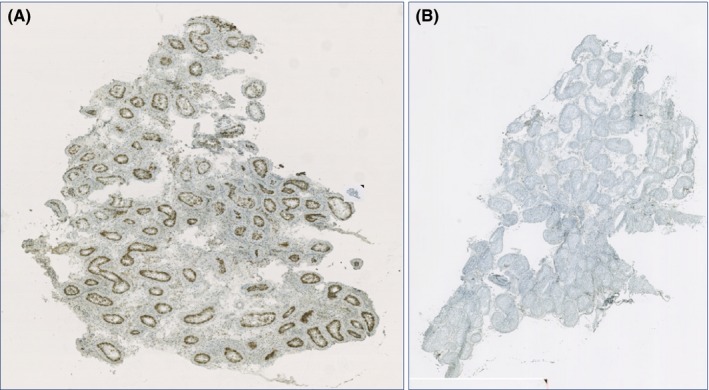
Cytokeratin‐18 (CK‐18), a marker for Sertoli cell immaturity, detected in the seminiferous tubules using a three‐stage immunoperoxidase technique as described in detail in the main text. Presence of CK‐18 was visualized using diaminobenzidine tetrahydrochloride (DAB). (A) A 29‐year‐old man treated twice with chemotherapy because of Hodgkin's disease, with Sertoli cells in a biopsy, positive for CK‐18. (B) A 36‐year‐old proven fertile man undergoing vasectomy, negative for CK‐18.

In the series of 33 azoospermic men and seven normal controls examined for CK‐18 (Fig. 5) and included in this project, CK‐18 was detected by immunohistochemistry in 10–100% of seminiferous tubules in five of 13 men without sperm production and in 20–90% of tubules in five of eight men with sperm production in 5–50% of tubules. This stands in contrast to only two of 12 men (CK‐18 in 5% and 50% of tubules) with sperm production in ≥90% of tubules and in 0 of the seven controls (*p* < 0.05). However, none of the 33 azoospermic men examined in this substudy showed THF at ultrasonography or TM at histological examination.

## Discussion

In this retrospective study of an unselected cohort of 382 non‐vasectomized, azoospermic men carefully examined by testicular ultrasonography during a systematic examination program, the prevalence of THF was 13.4%: pronounced in 5% and borderline in 8.4%. The prevalence of THF was particularly high in men with KS or a history of cryptorchidism. Remarkably, a major proportion of men with KS (14 of 44, ~32%) showed bTHF.

It may be suggested that THF may originate from degenerated intratubular germ cells not phagocytized by Sertoli cells the same way as TM as supposed by Vegni‐Talluri *et al*. (1980). However, not all hyperechogenic areas represent calcifications. Thus, the *Mediastinum testis*, consisting of connective tissue, vessels, veins, and nerves appear hyperechogenic, although not containing calcifications. Furthermore, microcalcifications were not detected histologically in testicular biopsies from any of the patients with ultrasonographically THF. Possible explanations for this might be that tissue sections for histological examination were cut between the THF/TM, or that microliths were lost during the fixing and staining procedures preceding histological examination. However, it seems unlikely that THF could in all case have been ‘hidden’, and as microliths were conserved at histological examination in ram testicles prepared in a way similar to the one used in this study (Fedder *et al*., 2016), it seems unlikely that the microliths should have been perished during preparation. Furthermore, cutting of testicular tissue with microcalcifications often cause foldings of the tissue sections (Fedder *et al*., 2016), which was not described in any prep in this study. Thus, THF not necessarily represent TM,

THF patterns occurring in stripes or beams (Fig. 3) might represent the testicular septae consisting of stripes of connective tissue with small vessels, veins, and nerves and dividing the testis parenchyma into testicular lobules.

It is a question why THF occurs with higher prevalence in men with cryptorchidism and KS, if THF represents connective tissue with vessels rather than proper testicular parenchyma. The higher prevalence of bTHF in men with KS might be related to the increased prevalence of vessel abnormalities observed in these (Tüttelmann *et al*., 2014). A higher rate of pronounced THF in men operated for cryptorchidism or having persisting cryptorchidism compared to men with milder forms of cryptorchidism may raise the hypothesis that TM in cryptorchidism is particularly associated with subgroups with severe cryptorchidism.

### Strengths and weaknesses of the study

The patient material was an unselected cohort of consecutively referred, azoospermic men.

As the included azoospermic men were not only examined clinically and with genetic and endocrinological analyses but the majority also histologically based on testicular biopsies, it was possible to compare the ultrasonographic findings with testicular histology, including malignancy and pre‐malignancy. Furthermore, it is a strength that all men were carefully examined by the same clinician using the same examination program and a 7.5 MHz linear ultrasound transducer during the entire study.

The weakness of this study is that the program was performed with the purpose to identify the etiology of the azoospermia and the treatment possibilities of the couple. Thus, the present results were not collected from a prospective study with THF defined as a specific outcome from the beginning. Nevertheless, testicular ultrasonography was systematically described for each testis during the primary examination of all men. Another weakness is that it was not possible to include a series of men with normal semen quality evaluated using the same THF criteria as a control group.

### Comparison with other studies

Different definitions of THF may complicate comparison of studies. When comparing studies, it is of central importance that identical definitions of THF are chosen. The predominant definition of >5 or ≥5 THF per transducer field is arbitrary. Similar to this study, Coffey *et al*. (2007) has classified THF according to: none, a limited, a scant, a moderate, or too numerous THF to count. Middleton *et al*. (2002) distinguished between classic and limited THF, the classic form representing pronounced THF as described here, while limited THF rather seems to be identical to bTHF.

The prevalence of THF has been assessed in two large series of healthy male volunteers recruited from the American and Turkish Army Reserve Officer Training Corps, 17–42 years old. In the US series, 5.6% of 1504 men showed THF (Peterson *et al*., 2001) compared to 2.4% of 2179 men in the Turkish series (Serter *et al*., 2006). The prevalence of THF is associated with ethnicity. Thus, Peterson *et al*. (2001) found TM in 14.1% black men compared to 4.2% in white men. The prevalence of THF was 2.4% in a Dutch study including 694 asymptomatic boys and young men 0 to 19 years old. The prevalence was significantly higher in the older boys compared to the youngest boys, suggesting that THF develops during childhood and puberty (Goede *et al*., 2009).

Several studies have demonstrated or suggested an association between THF and pathological andrological conditions such as KS (Martinez‐Valls *et al*., 2003 & Fedder *et al*., 2015), cryptorchidism (Nistal *et al*., 1979; Riebel *et al*., 2000 & Goede *et al*., 2010), and infertility (von Eckardstein *et al*., 2001 & Aizenstein *et al*., 1998). THF has been found strongly associated with the development of TGCN. In a metaanalysis including 33 papers, Tan *et al*. (2010) found a pooled risk ratio for concurrent TGCN of 8.5 (95% CI: 4.5–16.1) in the presence of THF in men with risk factors for TGCN. The pooled risk ratio for IGCN seems similar to the pooled risk ratio for TGCN (Tan *et al*., 2010), while the risk for TGCN and IGCN seems very low in adult asymptomatic men with THF but without risk factors (Peterson *et al*., 2001 & Serter *et al*., 2006). No clinical sign of testicular malignancy was observed in those 3683 men. Furthermore, 63 of 84 men with THF in the US study were included in a 5‐year follow‐up study, and only one man developed malignancy. Thus, a mixed germ cell tumor developed 64 months after the initial screening study (DeCastro *et al*., 2008). Therefore, THF is suggested not to be an etiological factor in TGCN – but rather a marker or a familial risk factor increasing the risk of TGCN (Coffey *et al*., 2007) particularly in men with other risk factors, for example cryptorchidism (Goede *et al*., 2010).

A positive point is that the majority of men with THF will never develop testicular cancer.

Therefore, an important question is how men with THF should be controlled and treated, depending on the presence of concomitant TGCN risk factors. When developing a ‘cost‐effective’ follow‐up program, it might be useful to distinguish between different THF patterns categorized as demonstrated in this study.

The cause–effect relations between THF and andrological conditions, including neoplasia, are not clearly highlighted. Coffey *et al*. (2007) found THF aggregated in families, not only in men with testicular neoplasia but also in unaffected male relatives. On this background, they suggest a common genetic susceptibility to THF and testicular neoplasia rather than considering THF as etiological factor for neoplasia.

Even though a high prevalence of THF is found in men with KS, these men usually have only few THF (Fedder *et al*., 2015), denominated as bTHF in this paper and discussed previously, and the incidence of testicular cancer in KS men is not higher than in other men (Hasle *et al*., 1995 & Swerdlow *et al*., 2005).

The cytoskeleton is composed of intermediate filaments, microfilaments, and microtubules. The intermediate filaments can be subdivided into six families, including the keratins described here and vimentin, which is the intermediate filament characteristic for normal adult Sertoli cells (Aumüller *et al*., 1992). In this study, we were able to detect CK‐18 in ~50% (10 of 21) of azoospermic men with impaired spermatogenesis, and without THF and TM, but only in two of 19 men with near normal spermatogenesis, showing that expression of CK‐18 in the Sertoli cells not necessarily cause THF. The mechanisms behind THF and TM may be complex.

In this study, remarkably high numbers of patients with uTHF and bTHF not only had a histological pattern with atrophy of the seminiferous tubules but also foci with production of spermatozoa or spermatids. It is exactly such azoospermic men with mixed testicular atrophy who show Sertoli cell immaturity demonstrated by expression of antimüllerian hormone (AMH) and cytokeratin‐18 (CK‐18) in their adult Sertoli cells (Maymon *et al*., 2000, 2002). Our data support that CK‐18 is more frequently expressed in testicles of men with mixed testicular atrophy. However, as none of the 33 azoospermic men examined for CK‐18 showed THF or TM, CK‐18 expression in Sertoli cells may be more frequent than presence of THF and TM.

## Conclusions and Future Research

A total prevalence of THF, pronounced or borderline, was retrospectively found in 13.4% and pronounced in 5% of 382 unselected, non‐vasectomized, azoospermic men. The relatively high frequency of bilateral uTHF and bTHF support the hypothesis of a general defective spermatogenesis as background of THF. THF seems more frequent than TM, and THF not necessarily represents TM.

The 19‐year‐old, azoospermic man, who had cryopreserved testicular spermatozoa from his one and only testis, illustrates the importance of alertness to cryopreservation of spermatozoa before treatment for GCN. In similar cases, it may be recommended to collaborate with a fertility clinic to optimize the ability of the patients to reproduce in later life. If the testis was primarily exposed to radiation, the fertility potential of this man would have been reduced to zero.

Several questions remain to be answered regarding a possible relationship between THF and reduced spermatogenesis as found in KS and cryptorchidism and the association between THF and testicular malignancy. When comparing studies, consensus according to definition of THF is a must. The commonly used limit of five THF in one transducer field is arbitrarily chosen. Ideally, all THF elements should be identified, localized, and counted, and the THF status of each testicle related to spermatogenesis, (pre‐)malignancy and etiology, and based on analysis of huge materials, a useful THF definition should be established. In addition to number and localization, attention may be given to each THF element. Thus, we have observed some more matte, grayish elements. Furthermore, larger prospective studies are needed to explain the mechanisms behind THF and to evaluate the predictive value of categories of THF for the presence of TGCN and IGCN in more details. Possible associations between testicular hyperechogenic loci and microlithiasis may be highlighted in animal studies.

## Disclosure

The author declares no conflict of interest.

## Authors' Contributions

JF has designed the study, carried out all clinical examinations including ultrasonography and testicular biopsy. JF has planned the immunohistochemical study and performed the blind readings of all samples. Finally, JF has written the manuscript.
